# A Fast-and-Robust Profiler for Improving Polymerase Chain Reaction Diagnostics

**DOI:** 10.1371/journal.pone.0108973

**Published:** 2014-09-30

**Authors:** George J. Besseris

**Affiliations:** Technology Management Department, City University of Seattle, Bellevue, Washington, United States of America; University of North Carolina at Charlotte, United States of America

## Abstract

Polymerase chain reaction (PCR) is an in vitro technology in molecular genetics that progressively amplifies minimal copies of short DNA sequences in a fast and inexpensive manner. However, PCR performance is sensitive to suboptimal processing conditions. Compromised PCR conditions lead to artifacts and bias that downgrade the discriminatory power and reproducibility of the results. Promising attempts to resolve the PCR performance optimization issue have been guided by quality improvement tactics adopted in the past for industrial trials. Thus, orthogonal arrays (OAs) have been employed to program quick-and-easy structured experiments. Profiling of influences facilitates the quantification of effects that may counteract the detectability of amplified DNA fragments. Nevertheless, the attractive feature of reducing greatly the amount of work and expenditures by planning trials with saturated-unreplicated OA schemes is known to be relinquished in the subsequent analysis phase. This is because of an inherent incompatibility of ordinary multi-factorial comparison techniques to convert small yet dense datasets. Treating unreplicated-saturated data with either the analysis of variance (ANOVA) or regression models destroys the information extraction process. Both of those mentioned approaches are rendered blind to error since the examined effects absorb all available degrees of freedom. Therefore, in lack of approximating an experimental uncertainty, any outcome interpretation is rendered subjective. We propose a profiling method that permits the non-linear maximization of amplicon resolution by eliminating the necessity for direct error estimation. Our approach is distribution-free, calibration-free, simulation-free and sparsity-free with well-known power properties. It is also user-friendly by promoting rudimentary analytics. Testing our method on published amplicon count data, we found that the preponderant effect is the concentration of MgCl_2_ (p<0.05) followed by the primer content (p<0.1) whilst the effects due to either the content of the deoxynucleotide (dNTP) or DNA remained dormant (p>0.1). Comparison of the proposed method with other stochastic approaches is also discussed. Our technique is expected to have extensive applications in genetics and biotechnology where there is a demand for cheap, expedient, and robust information.

## Introduction

The polymerase chain reaction (PCR) process progressively amplifies minimal copies of short DNA sequences in an economic and expedient fashion [1]–[5]. PCR has become a workhorse technique in genetic mapping, DNA sequencing and cloning [6]–[12]. Maximizing the amplification efficiency of a PCR process remains an unyielding challenge. Many PCR variants have been proposed that exploit the enzymatic activity of polymerase in vitro to dramatically increase the number of replicates for selected DNA fragments (amplicons). In all versions, the basic mechanism involves a repetitive cycling of denaturation, annealing and elongation of amplicons with primers. PCR applications support screening efforts in prenatal and parental testing, tissue typing, phylogenics, forensics, and oncogenics as well as in infection disease characterization and detection. High-quality PCR amplification performance relies on the drastic suppression of artifacts, bias and chimeras [13]–[15]. Artifacts are genes that did not exist in the start-up PCR mixture that, nevertheless, loom during the DNA fingerprinting process. Moreover, certain PCR process factors, if not optimally adjusted, tend to overturn the initial gene ratio causing bias. Chimeras primarily appear due to either template-switching in DNA formation or annealing partly-extended primers.

PCR process dynamics are reputed to be notoriously complex and application specific - innately interfering with the mechanism that regulates the amplicon count performance. Therefore, the main focus has been on maximizing amplicon count resolution from direct yet ‘quick-and-easy’ experimentation without relinquishing economic efficiency [16]–[18]. An ideal strategy for such an endeavor to be viable has to accomplish screening and fine-tuning of the examined controlling factors in a single step. The proposed technique should be harmoniously robust and assumption-free enabling the harnessing of the uncertainty for the fingerprinting process [19]–[22].

Cobb and Clarkson [23] and Caetano-Anolles [24] were among the first researchers that sought to borrow cost-effective ‘screening-and-optimization’ techniques from industrial quality control in order to improve DAF processes. Core feature was the implementation of Taguchi methods to design and translate small but dense datasets utilizing orthogonal arrays (OAs) [25]. Orthogonal arrays are special tools for planning smart trials. OAs are part of the broader area of fractional factorial designs (FFDs). FFDs are instrumental for the data design and generation stages in the domain of conducting scientific experiments – Design of Experiments (DOE) [26]. OAs are routinely used for minimizing resources and turnaround time in circumstances where either innovative experimentation or product/process improvement projects are in progress without meanwhile surrendering vital information. This tactic has also been experienced in areas less traditional in deploying structured OA-experimentation, such as for example in forensic science [27]. To reach to robust decisions, equally important is the analysis procedure for the OA-collected data in the DOE framework [28]. Implementation issues in DOE studies as well as their diverse applications in the fields of industry and engineering have been comprehensively researched [29], [30]. For applications in biotechnology in particular, there is also an extensive account about the strengths and the weaknesses of Taguchi-related DOE methods [31]. Recent studies provide a promising glimpse about how to optimize molecular assays for PCR processes in several circumstances that include investigations of venous thromboembolism, identification of Staphylococcus aureus and Clostridium perfringens alpha toxins as well as in general genotyping [32]–[35].

Exemplifying the pursuit for superior discriminatory power, the amplicon profile enhancement was treated with non-linear OAs for the epidemiological typing of *Pseudomonas aeruginosa*, in an arbitrarily-primed (AP) PCR procedure [36]. Through the implemented AP-PCR protocol, it was attempted to adjust four well-known controlling factors which included the concentrations of: 1) MgCl_2_, 2) dNTP, 3) a primer and 4) the DNA template. It is interesting that the researchers proceeded to completing their study by executing concurrently the two sequentially prescribed tasks - process screening and parameter optimization - in a single effort. In brief, process screening filters out weak influences from an initial group of investigated factors. Parameter optimization gears towards finding those optimal settings of the identified strong factors, such that the performance of the predicted response is maximized. The strategy of running the two sequential tasks concurrently commands agility in dealing with two intertwined outcomes which in turn is redeemed with delivering cheaper and faster results. As a concept it is not new to modern production operations, since it essentially mirrors solid reengineering tactics as recommended by stringent lean-engineering principles [48], [49].

In planning the recipes for the AP-PCR procedure, the above researchers were vigilant about the behavior for each individual influence in case not conforming to linearity. Therefore, they designed their trials with the provision to capture potential curvature trends if they were present. By implementing an L_9_(3^4^) OA, the four controlling factors were optimally programmed to saturation ensuring that each individual factor is tested at least on three settings – to uncover possible nonlinearity. Furthermore, it was decided that the scheduled experimental recipes not to be replicated in order to curtail dramatically the turnaround time and the associated costs for the study. Subsequent response (main-effects) graphs summarized the behavior of the four controlling factors in a practical manner [36]. In their report, the researchers concluded that all studied factors appeared to play some role in affecting the discriminatory power in the AP-PCR trials. The quality of their ensuing diagnosis was dependable on magnifying the resolution of the amplicon bands, thus allowing a greater dispersion of the detected polymorphism. Nevertheless, the reported profiles lacked of assigning any statistical significance on the outcomes. This is because standard techniques, such as analysis of variance (ANOVA) or general regression, cannot retrieve error contributions from saturated and unreplicated OA-planned datasets [37]. This paradox stems from the fact that all degrees of freedom gained from the conducted trials are exclusively distributed among the effects [38]. Consequently, no remaining degrees of freedom are available to form a pooled error for sizing the magnitude of the experimental uncertainty. Hence, the data translation step is interrupted prematurely producing no statistical significances while any computed descriptive statistics may only be assessed subjectively.

The selection of the AP-PCR design matrix for testing our new proposal on profiling the amplicon performance is two-fold:

1. It is the only study available in the literature inasmuch as a three-level, four-factor (non-linear) saturated-unreplicated OA is neatly utilized to screen and optimize amplicon resolution. This means that the reader may access through reference [36] the unique combination of a single series of nine executed PCR recipes along with visual evidence of their band distribution.
2. The AP-PCR method is technology for bacterial-strain DNA-fingerprinting that undisputedly introduces the largest complexity in the small OA-dataset when compared to its current competitor techniques. This is because the AP-PCR method is based on the amplification of random fragments through arbitrary primers and thus it constitutes a “low-tech” solution. The AP-PCR does not consolidate any prior knowledge of the targeted genome sequence. This fuzzifies intensely the profiling detectability for a collected OA-dataset. Thus, it may be considered among the most challenging PCR types to assess the predictive capabilities of the proposed profiler against complex ambience.

Additional concerns are raised when attempting to describe small-data designs, besides those that deal with the conditions of unreplication, saturation and non-linearity. Since non-linear OA schemes are remarkably compact by design, any source of contamination of the dataset might cause a propagation of severe errors across all investigated influences in a follow-up analysis [39]. Thus, an efficacious data converter should be capable of protecting the harvested information by confronting extremities with robust filters [40], [41]. Possessing a high breakdown point is imperative for a profiler of a small and dense dataset such that to recognize irregularities of unknown origin and then suppress them. The selection of the sample median features a robust location estimator with a maximum achievable breakdown point of 50%. It is an efficient and economical estimator because it requires merely ordering a group of observations. The fact that a method utilizes known reference distributions that possess superior power properties while resisting to surrender accuracy to adverse situations are aspects highly appreciated in profiling [42]. Finally, the method should be flexible and liberal enough to avert the entrapment that may be elicited by the sparsity assumption, i.e. the a priori restriction that not all of the examined effects are permitted to be either all weak or all strong [43]. A superb non-linear profiler should fend off variation leakage from the uncertainty term when gauging the strength for each particular effect. For the non-linear unreplicated-saturated OAs, this becomes of paramount importance because the variation due to the uncertainty retains a cryptic character in absence of any degrees of freedom.

The method we develop in this article is suitable for explaining stochastically non-linear saturated-unreplicated OA-datasets to be used for profiling concurrent tasks in a high-demand process, such as featured in the improvement of the AP-PCR performance. The technique promotes: 1) the decomposition of multi-factorials to corresponding single-effect surrogates, 2) the subsequent one-way contrasting for sizing the strength for each individual surrogate effect, and 3) a built-in detector for performing an internal-error consistency check. Using the novel surrogate response concept, the proposed method demonstrates that does not require the creation of new reference distributions. For the developments of the new ideas that will be stipulated in this article, we define as profiler, the screening (stochastic) device that allows the three-point tracing of an examined effect. Similarly, the meaning of extraction is congruent to the process of information harvesting [38]. Finally, in accord with the previous two conventions, the term “quantification” assumes the stochastic interpretation of determining uncertainty.

## Materials and Methods

### Method Outline

**Figure 1:**
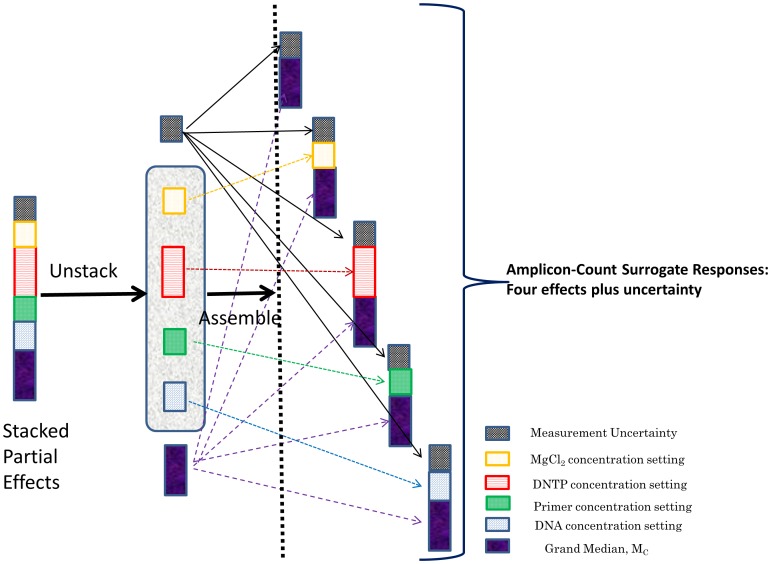
The configuration of a surrogate response by un-stacking and re-assembling an amplicon-count observation generated by the unreplicated-saturated OA scheme.

We outline ten steps to screen and optimize the amplicon count in a PCR process when data have been collected according to a non-linear unreplicated-saturated orthogonal array. The analysis stages proceed as follows:

1. Estimate the overall (grand) median for all amplicon data entries.
2. Estimate the setting medians for each effect separately.
3. Compute the partial (relative) effect due to each respective setting; it is defined as the difference of the setting median (Step 2) from the grand median (Step 1).
4. Estimate the error discrepancy for each observation by subtracting all respective partial effects (step 3) along with the grand median (step 1) from each measurement value.
5. Reconstruct the surrogate response entries by adding the respective partial effect and its corresponding error term to the grand median for each effect separately (Figure 1).
6. Form the surrogate error-vector entries by adding the respective error term to the grand median for each of the conducted trial runs separately (Figure 1).
7. Perform one-way non-parametric contrasting (Kruskal-Wallis test [45]) to each of the generated surrogate responses (Step 5). Repeat the comparison task for each effect individually using the surrogate error vector (Step 6).
8. Determine the important effects by inspecting the significance of their computed p-value (Step 6). Ensure that no effect is significant for the surrogate error contrasts (Step 7).
9. Select out the setting that maximizes the amplicon count for each of the strong effects individually.
10. Compiling the optimal partial responses for all strong factors (Step 9) on the grand median, predict the maximum value for the amplicon count. Form a 95% confidence interval by using the Wilcoxon test [41], [42] on the surrogate error vector. Confirm that the error is symmetrically spread across all observations.

### Computational Method

We consider the analysis of a collected amplicon-count (*C_A_*) dataset which has been planned by an L_9_(3^4^) OA to support a PCR screening-and-optimization study. The L_9_(3^4^) OA plan accommodates the manipulation of a maximum number of four controlling factors. Perturbing each examined factor at three distinct settings includes two endpoint settings that define the selected experimental range. The placement of an extra setting in between the two endpoints is imperative to simultaneously inquire about non-linear trends. A single execution of the L_9_(3^4^) OA requires gathering observations from nine predefined recipes. The nine resulting measurements comprise the unreplicated dataset which we conveniently symbolize as 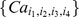 where each *i_j_* (*j* = 1, 2, 3, or 4) identifies the setting status of the *j* ^th^ influence. Thus, planning with the L_9_(3^4^) OA, there are only three admissible states appointed to each *i_j_*. In turn, each *i_j_* may be generically coded by assigning to it values such as ‘1’, ‘2’ and ‘3’, respectively. Usually, we reserve the indications ‘1’ and ‘3’ to denote the two endpoint settings. For the middle setting, we allot the coded setting ‘2’. Now, we propose an effects model that allows accounting for the experimental error. The error term, 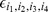, encompasses a random-error component along with any other spontaneous unknown and unknowable intrusions (if any). We may describe our proposed effects model as:
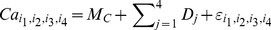
(1)We define in equation 1, the overall (grand) median, *M_C_*, of the nine amplicon-count observations as:
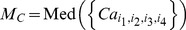
(2)The distinct combination of the four subscripts (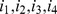), in equation 2, exactly mirrors the respective combinations of the four controlling factors in the nine recipes of the L_9_(3^4^) OA layout. Next, we define the median values of the amplicon-count response at their three respective factor settings, 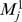, 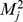 and 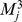 with 1≤*j*≤4. The setting measure, 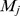, represents a median estimation of a group of observations that share the same factor setting *i_j_* (1≤*j*≤4):
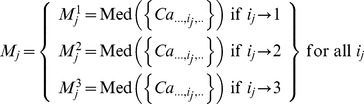
(3)Then, we define the indexed quantity *D_j_* (equation 1) as the difference between 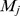 and *M_C_* which quantifies the *i_j_* ^th^ partial (relative) effect due to the *j* ^th^ factor with respect to the grand median:
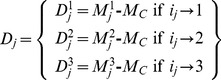
(4)After fitting equation 1, we dismantle the partial effect terms to form a surrogate (unstacked) response (Figure 1) for each effect separately which we denote as 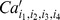. We reassemble a surrogate response by summing together: 1) the grand median, *M_C_*, 2) the partial effect, *D_j_*, and the corresponding error contribution, 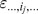 for all *i_j_*. The surrogate response acquires the following stochastic structure:
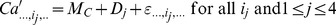
(5)For each controlling factor individually, we rank-order 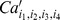 to transform it to a rank response, 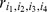:
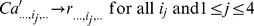
(6)We next form the mean rank sums for all three settings of the *j* ^th^ effect, 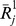, 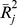 and 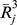:
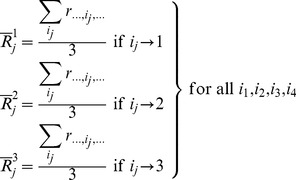
(7)Since the total number of observations across all settings for a given effect is N = 9, then, the Kruskal-Wallis test statistic, 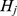 (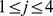), is written as:
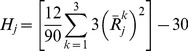
(8)To inspect the uniformity and stability of the residual error in the preceding ranking operations, we create a surrogate error response, 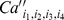. The surrogate error vector simply tracks how the error discrepancy for a given observation gyrates around the grand median value:
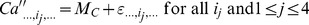
(9)Proceeding to rank-order the 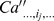 will yield the transformed response, 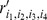:
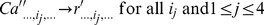
(10)Forming the mean rank sums of the 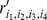 for all three settings of the *j* ^th^ effect, 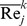, with *k* = 1,2 or 3, we obtain:
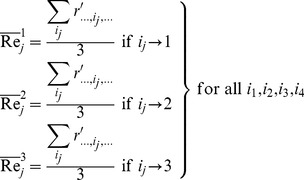
(11)The Kruskal-Wallis test statistic for the surrogate error is similarly defined as: 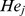, (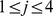):
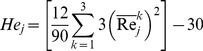
(12)The quantity 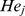 decodes potential intrusions in the dataset which could destabilize the validity of each observation. Thus, sharp spontaneous fluctuations of the error term could cause a negation of the significance of the screening results (equation 8). If the four calculated contrasts (equation 12) show that there is no statistical significant relationship between the four controlling factors and the experimental uncertainty, then, we may proceed to estimate a prediction for the optimized response. We identify the statistical significant effects from equation 8.

We select the optimal settings from all active effects, *m* (1≤*m*≤4), that maximize the magnitude of the amplicon-count median value. Utilizing equation 1, we calculate an estimation of the predicted response *Ca_p_* from the respective optimal partial effects, 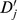:
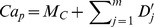
(13)We may compute the confidence interval for *Ca_p_* in equation 13 by performing the signed-rank (Wilcoxon) test for a single sample on the nine elements of the surrogate error vector, 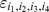. We prepare the main effects plot with the statistical software package MINITAB 16.2. The outcomes of the Bartlett's and Levene's tests for assessing the validity of the assumption for homogeneity of variances in an ANOVA treatment is also computed utilizing the MINITAB 16.2 software. The exact Kruskal-Wallis test significances are computed with the statistical software package SPSS 19.

## Results

**Figure 2:**
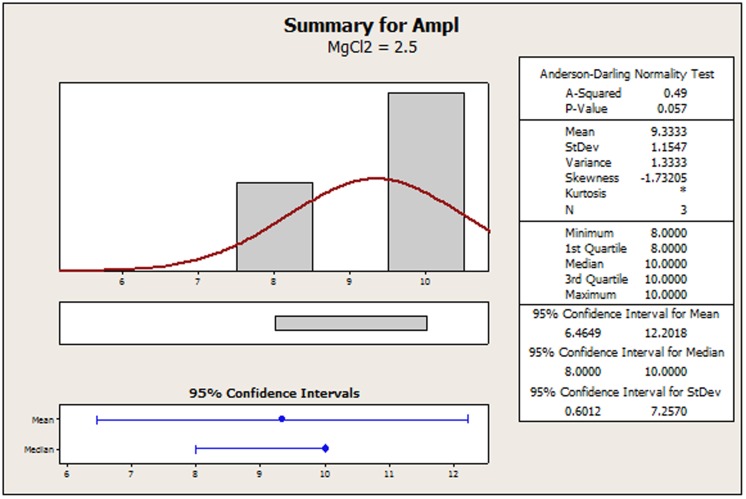
Summary statistics of amplicon performance for MgCl_2_ concentration at 2.5 mM.

**Figure 3:**
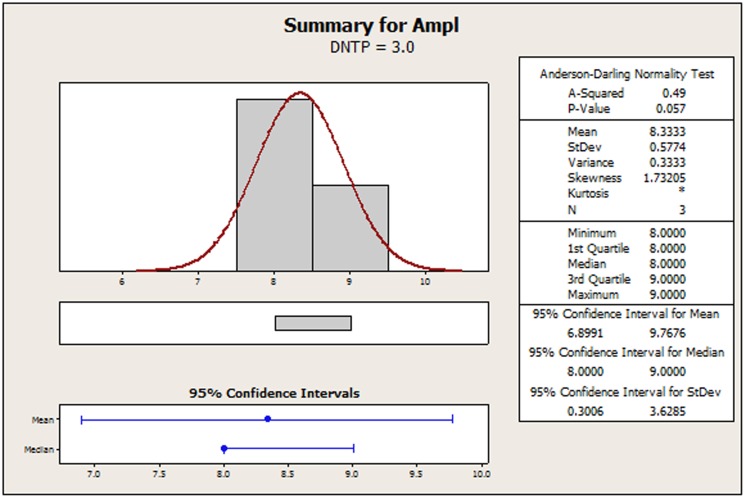
Summary statistics of amplicon performance for DNTP concentration at 3.0 mM.

**Figure 4:**
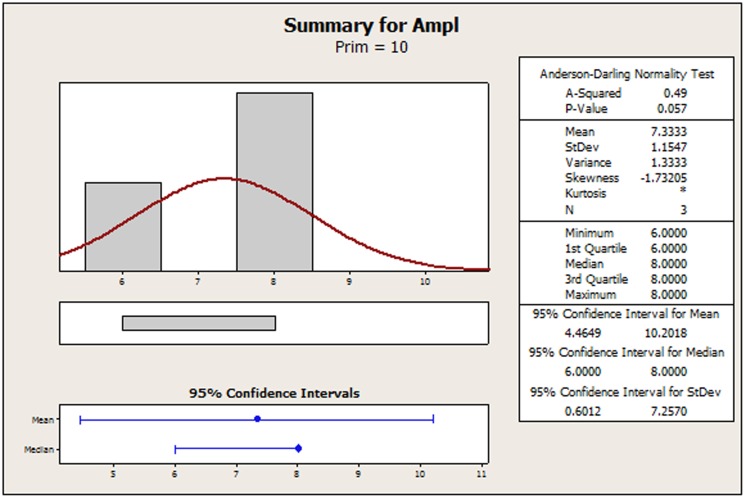
Summary statistics of amplicon performance for primer concentration at 10 pM/ µL.

**Figure 5:**
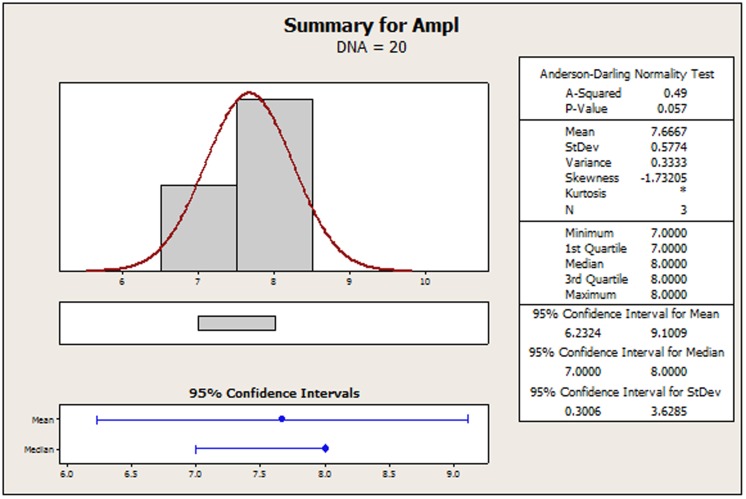
Summary statistics of amplicon performance for DNA concentration at 20 ng/ µL.

The original amplicon performance data for the AP-PCR procedure as it was acquired by Dabrowski et al. [36] have been tabulated in Table S1 for convenience. In Figure S1, we display a recreated main effects graph (MINITAB 16.2). Qualitatively, the concentrations of: 1) MgCl_2_, 2) the primer and 3) the DNA seem to perturb the amplicon response. It is the contents of MgCl_2_ and DNA that favor a strong non-linear tendency. Using Bartlett's or Levene's tests for checking the validity of the assumption for homogeneity of variances across all factor settings is of no avail because the setting samples are below the minimum requirement for both of those two tests. In Figures 2–5, we provide indicative graphical summaries of amplicon response for four selected factor settings. It is evident that a kurtosis estimation of the peakedness of the distribution within each factor setting will remain veiled in this profiling. Thus the profiler is forced to undergo the contrasting process while being deterred by a lack of information about the heaviness of the data distribution tails. Particularly puzzling is the switching of the skewness estimations from positive (Figures 2, 4, 5) to negative (Figure 3) probably implying the inverting of long tails for different factor settings. Skewness trends for all settings are listed in Table 1 to provide a more detailed view of the amplicon reaction to different controls. The most striking event is attributed to the primer content which spurs on all three possible outcomes. Indeed, skewness abruptly transverses from short to long tails as the primer content increases and then at the upper endpoint suddenly balances out to symmetry (skewness = 0). From Table 1, we observe that only 33% (four out of twelve) of the settings may be prone to symmetry. This may predispose to the existence of different underlying mechanisms – with varying distributions - that might irregularly manipulate the amplicon output. Thus, the presence of any predominant data symmetry may be debated as it is also supported by the median location on the 95% confidence interval portrayed in the four respective summaries (Figures 2–5). Depicting the central tendency of the factor settings in terms of the median estimator is justified because it yields a 95%-confidence interval with a tighter outlook than the mean statistics version could offer. The above findings are exacerbated by noticing that the Anderson-Darling test allows the rejection of the normality hypothesis at an error rate of 0.1 for all four displays (Figures 2–5). This translates to an observation that 33% (four out of twelve) of the setting effects exhibit an indeterminate character that substantially deviates from normality. Lastly, the amplicon performance with respect to the concentration of MgCl_2_ set at 2.0 mM could not be analyzed at all since all related values in the output column are identical. Therefore, it is hard to discern if there are inherent anomalies in the evolving phenomena or it is a manifestation of multiple superimposed mechanisms that blur the stochastic blueprint of the amplicon response.

**Table 1:** Skewness of the amplicon OA-data for the AP-PCR study.

Factor	Setting	Skewness
**MgCl_2_**	**(mM)**	
	2	0.94
	2.5	−1.73
	3	*
**DNTP**	**(mM)**	
	1.5	0
	2	0.94
	3	1.73
**Prim**	**(pM/ µL)**	
	10	−1.73
	20	0.94
	30	0
**DNA**	**(ng/ µL)**	
	10	0
	20	−1.73
	30	0

*Incalculable.

Un-stacking and reconfiguring the AP-PCR amplicon-count experiments generate five surrogate vectors (Table 2). Visual inspection reveals that from the tabulated surrogate vectors it is C_MgCl2_, C_Prim_ and C_DNA_ that carry the largest fluctuation in the ranked entries which numerically vary from magnitudes of 6 to 9. The amplicon response should be maximized for better resolution. We notice that in the C_MgCl2_ column the frequency of entries scoring a rank of 9 is double with regards to the other two surrogates. This should give MgCl_2_ concentration a lead over the rest of the group. We discover that only the MgCl_2_ concentration is statistically significant to a level of 0.05 (Table 3). The primer concentration appears to be potent if we are willing to accept a cruder refinement at a significance level of 0.1. We infer from Table 3 that the concentrations of the DNA template and dNTP should drop out as statistically dormant influences (p>0.1). It is also noteworthy to report that the fluctuation owing to the uncertainty component balances evenly across all factor settings (p>0.1). This result supports the validity of the hierarchical status of the two active factors found above. Concluding with the screening phase, we proceed to fine-tuning the two influencing factors. From Table 3, we read off the optimal settings that favor the maximization of the amplicon count. The median amplicon count achieves a value of 10 when the concentration of MgCl_2_ is 2.5 mM. Similarly, the primer concentration set at 30 pM/µL aids in sustaining the amplicon count to 9 bands. Since our proposed model is additive, the predicted amplicon value which includes the contributions from both active factors will be: 10 ( = 8+2+1+(−1)) with a 95% confidence interval of [10], [11]. The false discovery rate has been controlled in this study at a reasonable value of q* = 0.2 [44].

**Table 2:** Rank-ordered amplicon data*.

Run #	C_MgCl2_	C_DNTP_	C_Prim_	C_DNA_	C_e_
**1**	6	7	7	7	7
**2**	7	8	8	8	8
**3**	7	8	9	9	8
**4**	9	7	7	8	7
**5**	9	7	8	7	7
**6**	8	6	6	6	6
**7**	7	7	8	7	7
**8**	7	7	7	8	7
**9**	8	8	8	8	8

* C_MgCl2_: surrogate response for MgCl_2_ concentration.

C_DNTP_: surrogate response for DNTP concentration.

C_Prim_: surrogate response for primer concentration.

C_DNA_: surrogate response for DNA concentration.

C_e_ : surrogate response for uncertainty.

**Table 3:** Nonparametric Response Table*.

Factor	Setting	Median C_A_	Effect Significance	Error Significance
**MgCl_2_**	**(mM)**			
	2	7		
	2.5	10		
	3	8	**0.043**	0.357
**DNTP**	**(mM)**			
	1.5	8		
	2	8		
	3	8	0.679	0.679
**Prim**	**(pM/µL)**			
	10	8		
	20	8		
	30	9	**0.064**	0.357
**DNA**	**(ng/µL)**			
	10	8		
	20	8		
	30	9	0.257	1.000

*Exact Kruskal-Wallis test-statistics results.

## Discussion

We presented a novel assumption-free technique for dealing with dense datasets suitable for profiling effects with potentially curvature tendencies such as in an AP-PCR procedure [36], [50]. To avoid confronting directly the pooled-error determination, we proposed an additive non-linear model for screening saturated-unreplicated OA-data [37]. We built our model around a pivotal baseline (grand median) where the partial effects may be stacked atop each other while granting an uncertainty term. Such a model facilitates the decomposition of a densely-compacted dataset during the information extraction phase [38]. We defined the partial effect at a given setting to be the disparity of each effect's median estimation from the baseline value. By slicing each observation to its constituent parts, we un-stacked and reconstructed the decompressed responses. The behavior of the surrogate effects is reflected through the engineered standalone responses. We re-assembled each surrogate-response entry for each of the collected observations by adding together three quantities: 1) the baseline, 2) the partial effect contribution, and 3) the uncertainty which is tagged to the corresponding trial run. We reserved a separate surrogate response for uncertainty by simply retaining the error term around the grand median. The uncertainty surrogate is a mandatory response for checking the uniform stability of the behavior of the error terms across all factor settings. This last action is unavoidable because of the experimental recipes not being replicated. We exchange the inability to locate a single central tendency for the experimental error with an assurance check which could track down any intrusion not spreading evenly across the executed recipes. Otherwise, the effect predictions are bound to be misleading. By decompressing the stacked effects to individual surrogate responses, we isolate the reconstructed datasets such that to be adapted each time for a single-factor comparison treatment with the well-known Kruskal-Wallis test [45]. The Kruskal-Wallis family of reference distributions has been studied extensively in the past. Moreover, the Kruskal-Wallis test has been categorized to complement robust comparison techniques with known power and efficiency properties [46]. Thus, contrasting outcomes have immediate impact not requiring extra calibration or simulation work [38]. Additionally, our technique does not presume that a subgroup of the studied effects should be necessarily weak, thus, it is not limited from the sparsity condition [26], [39].

We bypass the requirement for explicit error variance estimation but still managing to assign statistical significance to each of the studied effects. For the elucidated AP-PCR case, we only needed to contrast separately each of the four surrogate responses at their three respective settings while checking the behavior of the uncertainty response across the four factors for consistency [36]. Discovering statistically significant relationships while engaging the uncertainty (surrogate) response with respect to any of the examined effects could negate the decision about the potency of that effect. Such anomalous relationships could occur if specific surrogate response entries receive favorable stochastic ordering not because of the elicited effect but because of the size of unknown and unknowable intrusions lurking in the measurements [38]. Therefore, the proposed method possesses internal error-checking properties which decode the strength of the perturbing uncertainty. In lack of replicated data, the proposed technique undertakes a search for clues from recipe to recipe in order to spot inconsistencies and extremities with respect to the size of the uncertainty. The new approach encourages the efficient use of the information content in each single observation appreciating the aspect that unreplicated OA datasets are scarce and thus precious resources for knowledge discovery.

Direct competing non-linear techniques that incorporate screening and optimization in a single step for saturated-unreplicated OA schemes are still in the developmental stage [38]. To realize the usefulness of the proposed technique, we tabulated the corresponding ANOVA and GLM outputs in Tables 4 and 5, respectively. We observe that in both treatments the data processing has been aborted hastily due to the disappearing of any remaining degrees of freedom that could be associated to the experimental error. The only qualitative information we may extract from Table 4, for instance, it might be that the MgCl_2_ and the primer concentrations should lead the strength hierarchy in the examined group of effects. But the statistical importance of such an outcome cannot be quantified [26]. Freeing up some degrees of freedom which have been previously awarded to the effects may permit the statistical estimation of the experimental uncertainty. This tactic was suggested through the error-pooling approach found in the standard Taguchi-methods toolbox [25]. Nevertheless, a convenient error-pooling maneuver merely seeks to dislodge the weakest performing effect while disguising it instead as an entrapped quantity posing as the residual error. This is usually accomplished by identifying first and then removing the weakest effect from the initial list of the contrasted factors in the ANOVA treatment. The isolated variance of the weakest effect then enters the F-test comparison step in ANOVA playing the role of the unexplainable error [28]. Thus, this trick enables ANOVA to return estimations of statistical significance for the rest of the examined effects in the group by lifting the roadblock of the indeterminate uncertainty in connection with the depleted degrees of freedom. Generating ANOVA results in this fashion is still viewed as greatly subjective because the unexplainable error is rendered: 1) quantized and 2) framed to the size of the disturbance caused by the weakest effect [38]. Therefore, it becomes debatable whether the contribution of the uncertainty should be allowed to be limited to absorb only the weakest effect. Thus, the decision still looms with regards to what extent would be justifiable for other weaker effects to join in forming the residual error term. Similar discussion follows from using GLM regression to quantify the dominant effects (Table 5).

**Table 4:** ANOVA results for the PCR data of epidemiological typing of *Pseudomonas aeruginosa* above (MINITAB 16.2).

*Source*	DF	Seq SS	Adj SS	Adj MS	F-ratio	p-value
**MgCl_2_**	2	6.22	6.22	3.11	*	*
**DNTP**	2	0.22	0.22	0.11	*	*
**Prim**	2	4.22	4.22	2.11	*	*
**DNA**	2	2.89	2.89	1.44	*	*
Residual Error	0	*	*	*		
Total	8	13.56				

*Not calculable.

**Table 5:** General Linear Model results for the PCR data of epidemiological typing of *Pseudomonas aeruginosa* above (MINITAB 16.2).

Term	Coefficient	SE Coefficient	t-value	p-value
*Constant*	−37.000	*	*	*
*Prim*	0.150	*	*	*
Prim*Prim	−0.002	*	*	*
*MgCl_2_*	34.000	*	*	*
MgCl_2_*MgCl_2_	−6.670	*	*	*
*DNA*	−0.280	*	*	*
DNA*DNA	0.008	*	*	*
*DNTP*	2.220	*	*	*
DNTP*DNTP	−0.440	*	*	*

*Not calculable.

**Figure 6:**
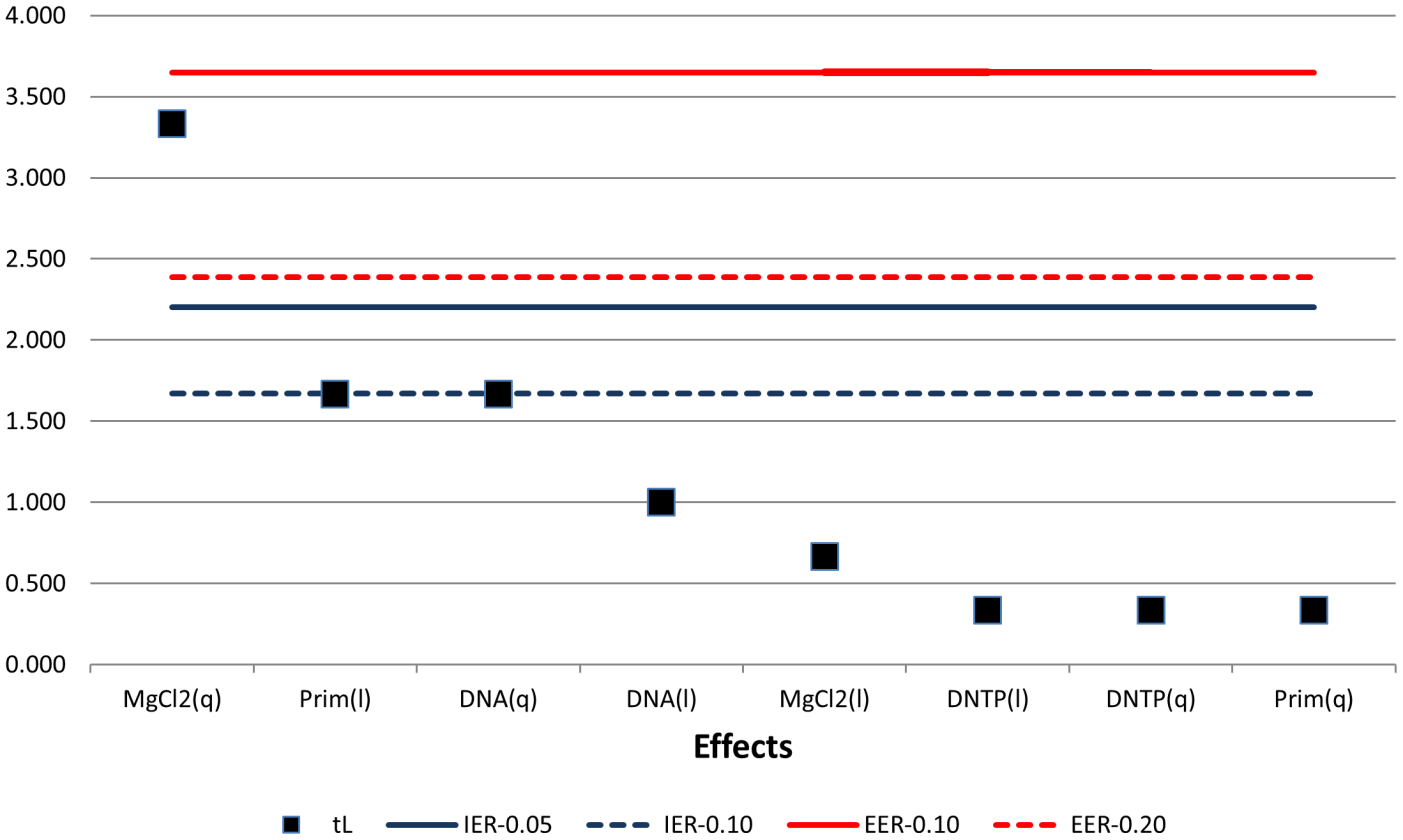
Lenth test profiling results using the corrections of Ye and Hamada [**47**].

Alternatively, the non-linear gauging of the effects may be approximated by dichotomizing each contrast first in linear and quadratic components in order to set them up appropriately for treating them with the Lenth test [47]. However, in such case the effects are diluted before they are fed to the data analyzer. This is because the effects do not participate as a single entity, but on the contrary they appear to possess a split identity. The subsequent inflation of the members of the tested group with virtual effects tampers with the process of extracting reliable statistical significance. This may be observed from Figure 6 where the effects are depicted in terms of their linear (l) and quadratic (q) components. At an experimentwise error (EER) of 0.2, the non-linear part of the MgCl_2_ content solely stands out as a viable influence which is also recovered from an individual error rate (IER) of 0.05. This virtual doubling of the actual number of the participating effects seems to instigate the depression of the predicted influence of the primer concentration. This is owing to the dependence of the number and size of the participating effects in calculating the pseudo standard error (PSE) in the Lenth test. The value of PSE was computed to be 2.12 for the AP-PCR example. In Figure 6, the corrected t-statistic quantity for each effect [47], t_L_, is stacked against the two ordinary limits for goal-posting the IER; they are drawn at error rates of 0.05 and 0.1, respectively. Clearly, only the quadratic part of the MgCl_2_ content makes the cut at the refined error rate of 0.05. On the same figure, the EER limits have also been drawn at error rates of 0.1 and 0.2, respectively. The only effect that makes the cut at the error rate of 0.2, but fails at the error rate of 0.1, is again the quadratic term of the MgCl_2_ content. Therefore, the calibrated Lenth test discounts the efficacy of the primer concentration succumbing to a type II error.

Using the non-parametric composite approach [43] to test the validity of our results for the AP-PCR study is anticipated to become a cumbersome task. This is because the family of reference distributions that could accommodate two different groups of tied observations - including one group that contains four ties at a particular value - has not been published for the composite screening of a saturated L_9_(3^4^) OA-dataset. Thus, additional combinatorial computations are needed to scale the ordered data. Immediately, we recognize the crucial advantage of flexibility instilled in the proposed method over the nonparametric composite screening method. This feature relieves the experimenter from accessing specialized routines to program extra simulations adapted each time to the specific arrangement of the ties in the dataset column. On Table 6, we present the rank-ordering of the original AP-PCR OA-dataset. Based on those rankings, we prepare the nonparametric response table (Table 7) for testing at least the existence of a single predominant effect with the composite method. The Kruskal-Wallis H-statistic corrected for ties is tabulated on Table 7 assorted with its corresponding exact p-value for each effect separately. It appears that no effect surfaces as a single standout utilizing the one-factor composite method. This demonstrates that even in the case we filter individually for the dominance of a single effect, the composite screening method proves also to be substantially less sensitive than the proposed method when we intend to control for IER at the customary levels of 0.05 and 0.1, respectively.

**Table 6:** Rank ordering of the original AP-PCR OA-data (Table S1).

MgCl_2_	DNTP	Prim	DNA	rAmpl
2	1.5	10	10	1
2	2	20	20	2
2	3	30	30	7
2.5	1.5	20	30	8.5
2.5	2	30	10	8.5
2.5	3	10	20	4.5
3	1.5	30	20	4.5
3	2	10	30	4.5
3	3	20	10	4.5

**Table 7:** Kruskal-Wallis statistics for the orderings in Table 6.

Factor	Setting	SR*	H (p-value)
**MgCl_2_**			
	2	10	3.4 (0.22)
	2.5	21.5	
	3	13.5	
	**SSSR** **	**744.5**	
**DNTP**			
	1.5	14	0.01 (1.0)
	2	15	
	3	16	
	**SSSR** **	**677**	
**PRIM**			
	10	10	2.45 (0.35)
	20	15	
	30	20	
	**SSSR** **	**725**	
**DNA**			
	10	14	2.06 (0.46)
	20	11	
	30	20	
	**SSSR** **	**717**	

*SR: Sum of Ranks.

**SSSR: Sum of Squared SRs.

The potential of our approach is ostensibly unlimited for speedy and cheap profiling in genetics and biotechnological applications at large. This is because it demands no knowledge about the detailed mechanism of a parametric model which often involves hard-to-validate reference distributions. It merely requires the establishment of a simple input-output relationship among the effects and the examined characteristic. It is also user-friendly by promoting rudimentary analytics.

The strong non-parametric character of our approach alienates the solver maneuverability from antecedent knowledge of the host reference distributions which are engrained each time by different genotyping conditions. Consequently, this last feature renders our methodology superbly adaptable for interpreting qPCR processes as well as specific multiplex-PCR datasets [50]. Thereby, our approach may be seamlessly implemented for deciphering complex genomics-responses such as the limit of detection along with the amplification efficiency. With regards especially to the maximization of the amplification efficiency which is usually anticipated to surpass in some circumstances the typical limit of 100%, the proposed approach may offer the only alternative. This is because percentage data (*p* in %) are commonly converted by the omega transformation {Ω(dB) = 10·Log_10_[*p*/(100-*p*)]}, before they are analyzed statistically with a multi-factorial application [25], [28]. The omega function tends to infinity as percentage data values approach either limits of 0% or 100%. Thus, for *p*>100%, the omega function computations halt as the logarithm of the resulting negative odds {*p*/(100-*p*)<0} is not meaningful. On the other hand, our approach which is based on primitive rank-ordering statistics is not inhibited by such a dataset idiosyncrasy.

## Conclusions

The performance of a polymerase chain reaction is sensitive to suboptimal processing conditions leading to artifacts and bias that eventually downgrade the discriminatory power and reproducibility of the results. Popular unreplicated-saturated orthogonal-array schemes have been implemented in the past to conveniently program and profile the non-linear amplicon response against unharnessed background noise. A novel assumption-free analyzer has been developed and tested on dense L_9_(3^4^) OA-data to investigate how to systematize the maximization of the amplicon performance. The profiling process was materialized by dissecting uncertainty and confirming its uniform manifestation across all conducted trials by introducing the concept of surrogate responses. Synchronously, the data conversion step retrieved the optimal settings of the dominant effects after probing and filtering out the negligible influences. The novelty of the method rests on its capability to glean statistical significance through the utilization of non-linear robust analytics directly on isolated surrogate responses. The underlying agility to circumvent the necessity for quantifying a residual error also upholds the resiliency to any stealth intrusions. Subsequently, effect sizing was simplified by using the distribution-free reference scale of Kruskal-Wallis for the inference effort. Moreover, AP-PCR diagnostics may be interpreted without requiring voluminous simulations or being restricted to inelastic assumptions attributed to data normality, variance homogeneity, effect dilution and effect sparsity. The technique is shown to possess superior efficiency and effectiveness when compared to alternative profiling strategies such as the composite nonparametrics and the corrected Lenth method. For the illustrated amplicon maximization problem, two controlling factors were identified as dominant at an experimentwise error rate of 0.2 - the concentrations of: 1) MgCl_2_ and 2) the primer. At an individual error rate of 0.05, the only predominant factor was the concentration of MgCl_2_. The predicted amplicon performance tops a value of 10 bands when the concentration of MgCl_2_ and the concentration of the primer are set at 2.5 mM and 30 pM/µL, respectively.

## Supporting Information

Figure S1 **The main effects plot for the PCR data of epidemiological typing of** ***Pseudomonas aeruginosa*** **from Table S1 (MINITAB 16.2).**
(TIF)

Table S1 **Original non-linear amplicon-count data for PCR profiling of epidemiological typing of** ***Pseudomonas aeruginosa*** **** [**36**] **.**
(DOCX)
