# Supplementary figures and images for: A Fast-and-Robust Profiler for Improving Polymerase Chain Reaction Diagnostics
Source: PLoS One. 2014 Sep 30;9(9):e108973. doi: 10.1371/journal.pone.0108973 (PMC4182614; doi:10.1371/journal.pone.0108973)

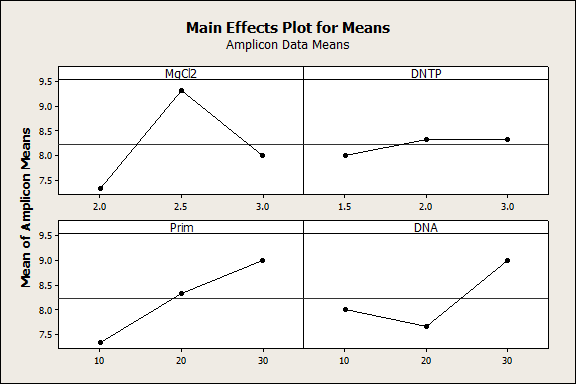

Supplement: Figure S1 — The main effects plot for the PCR data of epidemiological typing of Pseudomonas aeruginosa from Table S1 (MINITAB 16.2). (TIF) [file pone.0108973.s001.tif]
