# Supplementary material for: A Fast-and-Robust Profiler for Improving Polymerase Chain Reaction Diagnostics
Source: PLoS One. 2014 Sep 30;9(9):e108973. doi: 10.1371/journal.pone.0108973 (PMC4182614; doi:10.1371/journal.pone.0108973)
Supplement: Table S1 — Original non-linear amplicon-count data for PCR profiling of epidemiological typing of Pseudomonas aeruginosa [36] . (DOCX) [file pone.0108973.s002.docx]

| Run # | MgCl_2_ (mM) | DNTP (mM) | Prim (pM/μl) | DNA (ng/μl) | Ampl (No. of bands) |
| --- | --- | --- | --- | --- | --- |
| 1 | 2 | 1.5 | 10 | 10 | 6 |
| 2 | 2 | 2 | 20 | 20 | 7 |
| 3 | 2 | 3 | 30 | 30 | 9 |
| 4 | 2.5 | 1.5 | 20 | 30 | 10 |
| 5 | 2.5 | 2 | 30 | 10 | 10 |
| 6 | 2.5 | 3 | 10 | 20 | 8 |
| 7 | 3 | 1.5 | 30 | 20 | 8 |
| 8 | 3 | 2 | 10 | 30 | 8 |
| 9 | 3 | 3 | 20 | 10 | 8 |
